# Profunda Femoris Pseudoaneurysm following Total Hip Arthroplasty Revision

**DOI:** 10.1155/2015/301949

**Published:** 2015-08-10

**Authors:** Katharine Harper, Justin Iorio, Easwaran Balasubramanian

**Affiliations:** Department of Orthopedic Surgery, Temple University Hospital, 3401 N Broad Street, Philadelphia, PA 19140, USA

## Abstract

Vascular injuries following total hip arthroplasty (THA) are very rare, with pseudoaneurysm being a small subset. We report a case of profunda femoris artery (PFA) pseudoaneurysm in a 61-year-old male following a posterior approach revision left THA. Presentation involved continued blood transfusion requirements several weeks postoperatively. Diagnosis of the pseduoaneurysm was made by contrast CT of the lower extremity, with confirmation via IR angiography. Successful embolization was achieved with selective coiling and Gelfoam. Presenting complaints of such complications are often vague and therefore lead to delayed diagnosis. Causes of such complications are not completely understood, particularly with PFA injuries in THA. Possible mechanisms are discussed in this paper. Vascular complications following THA can be difficult to diagnose. High suspicion in the setting of continued postoperative pain or bleeding may allow prompt diagnosis and avoidance of serious limb-threatening complications.

## 1. Introduction

Total hip arthroplasty (THA) is one of the most commonly performed orthopedic procedures, which have consistently relieved pain and improved function in patients [1]. The procedure has a low complication rate of 6% [2], where the incidence of vascular injury is even lower [3]. However, vascular injuries may lead to catastrophic complications, including perioperative bleeding or critical limb ischemia [4]. Vascular injuries occur at a reported rate of 0.25% of all complications following hip surgeries [3]. Pseudoaneurysms are a very rare complication in the subset of vascular injuries.

We present a case of a profunda femoris artery (PFA) pseudoaneurysm following THA revision surgery. Profunda femoris aneurysm has only been reported a handful of times in recent literature and never from a posterior approach for a revision THA.

## 2. Case Presentation

**Figure 1:**
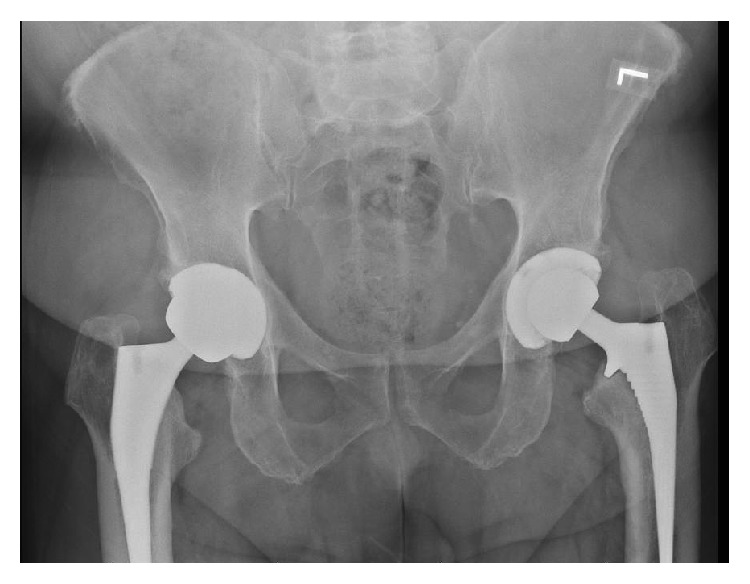
AP pelvis of the primary THA showing good alignment without fracture.

**Figure 2:**
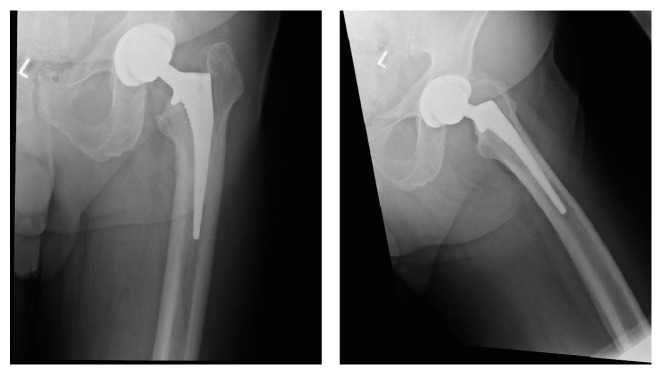
AP and lateral of the primary THA showing good alignment without fracture.

**Figure 3:**
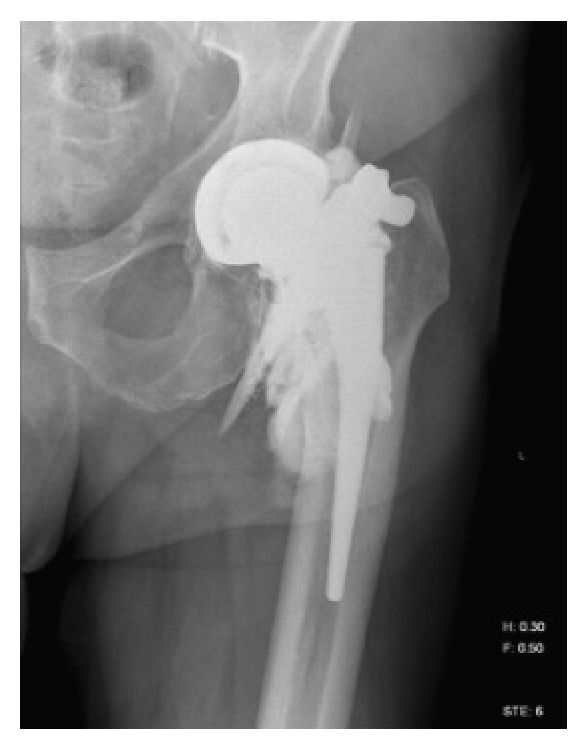
X-ray arthrogram showing mild contrast tracking along the proximal lateral aspect of the femoral component.

A 61-year-old male presented with complaints of pain in his left hip following an anterior approach for a THA, which was performed 8 months earlier at an outside institution. Per the operative report, there were no complications encountered with the surgery, including no femoral cortex perforation during reaming. He had undergone appropriate rehabilitation following the index procedure, but his symptoms remained refractory to analgesics and physical therapy. Upon arriving to our practice, a workup was begun for septic loosening of the femoral and/or acetabular component. Initial radiographs at our practice show well-aligned femoral and acetabular component without obvious evidence of loosening and with no periprosthetic fracture (Figures 1 and 2). Appropriate laboratory tests, including hip aspiration arthrogram, excluded infection as the etiology of his symptoms. A bone scan demonstrated hyperemia in the left thigh and increased uptake surrounding the femoral prosthesis. A subsequent X-ray arthrogram showed mild extravasation of contrast to the proximal aspect of the femoral component (Figure 3), suggestive of aseptic loosening of the femoral stem.

**Figure 4:**
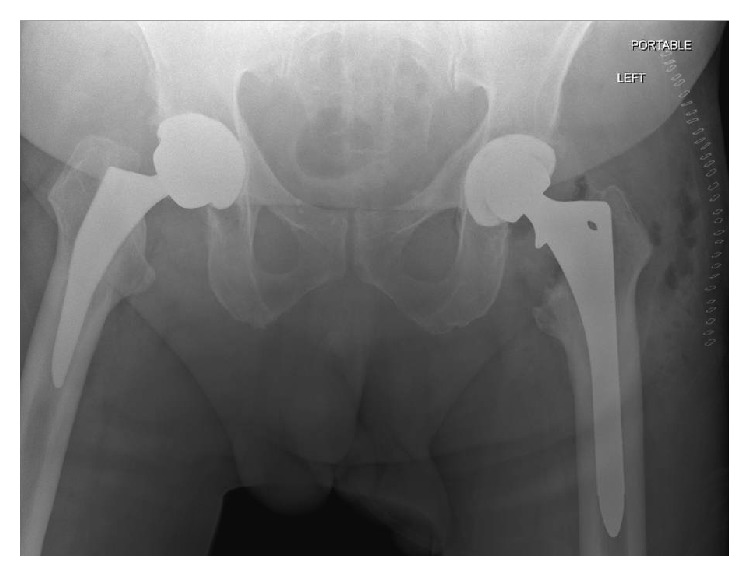
AP pelvis postoperatively showing good alignment without fracture or femoral cortex perforation.

**Figure 5:**
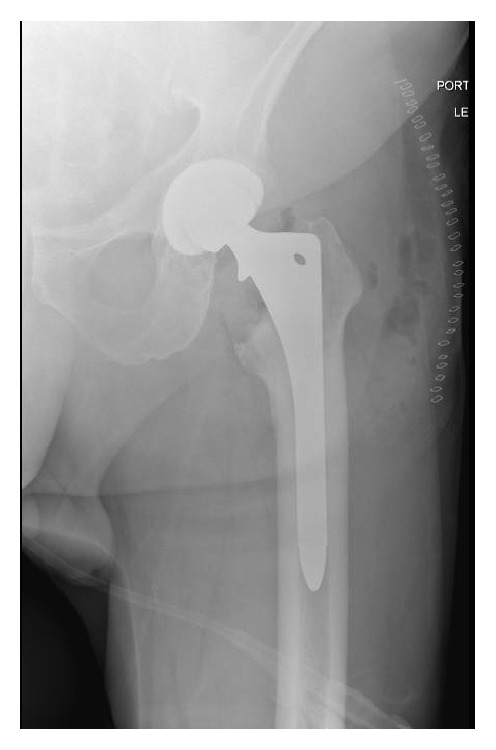
AP hip postoperatively showing no fracture or femoral cortex perforation.

The patient underwent revision of the femoral stem for THA without immediate complication. A standard, posterior approach was performed and blunt, Hohmann retractors were placed anteriorly and medially around the proximal femur for exposure of the femoral canal for reaming and cement removal. There were no complications encountered intraoperatively. Postoperative radiographs show the femoral and acetabular components in good positioning without periprosthetic fracture or femoral cortex perforation (Figures 4 and 5). He was discharged in stable condition on the third postoperative day to a skilled nursing facility.

10 days postoperatively he presented to the emergency room for tachycardia. Workup in the ER included INR (3.6), basic labs, and a CT of his left lower extremity. CT showed concern for possible underlying hematoma in the operative field. He was admitted for symptomatic anemia (Hb, 7.9 mg/dL). On hospital day 1, his hemoglobin dropped to 6.9 mg/dL and he underwent transfusion of packed red blood cells. His Coumadin was discontinued because of the concern for hematoma. Following this transfusion, his hemoglobin stabilized at 8.2 mg/dL and he was discharged on hospital day 4 to his skilled nursing facility.

He presented 4 weeks later for routine follow-up at which time he was progressing well.

**Figure 6:**
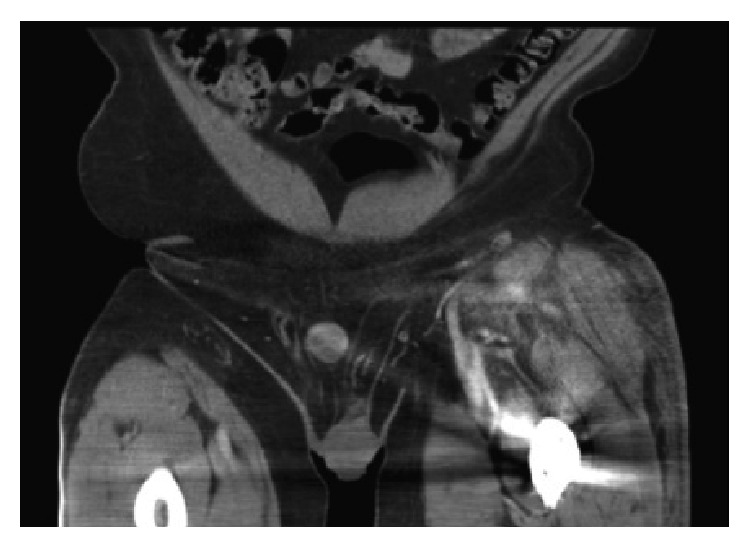
Contrast CT of the left lower extremity showing a suspected pseudoaneurysm of the left profunda femoris artery.

**Figure 7:**
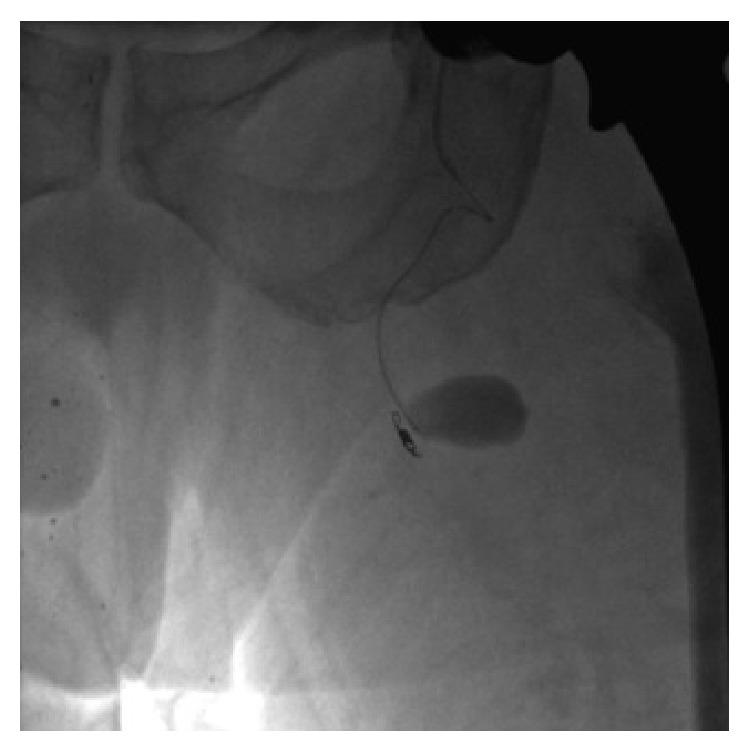
IR angiogram demonstrating 13 mm pseudoaneurysm of the profunda femoris artery.

**Figure 8:**
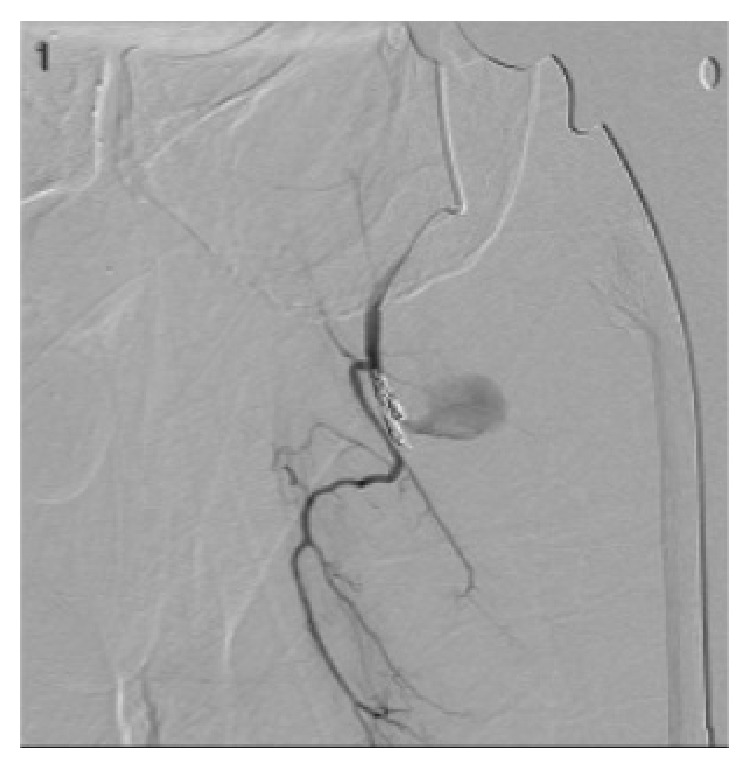
Imaging of embolization of the pseudoaneurysm by coiling and Gelfoam, performed by IR.

**Figure 9:**
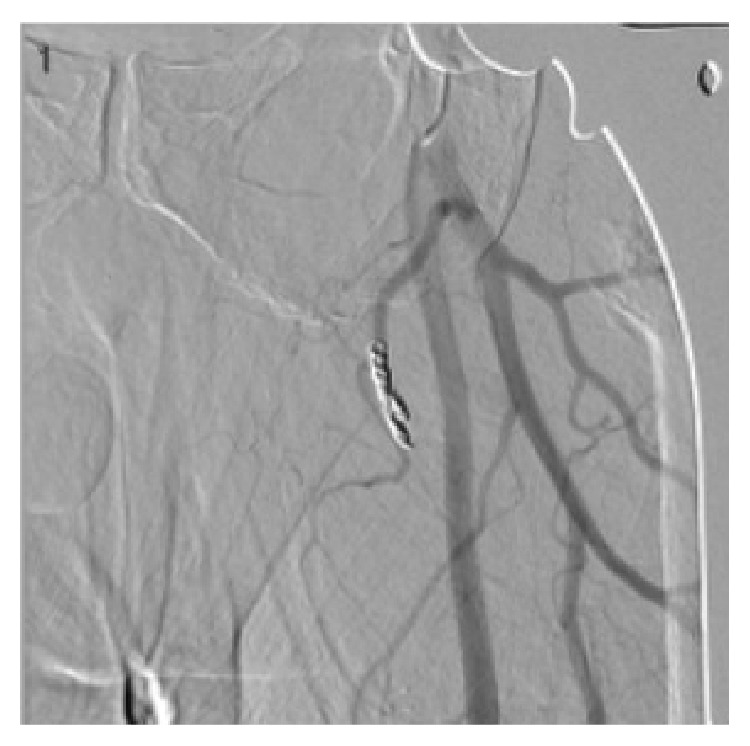
Angiogram showing coil in profunda femoris artery with elimination of the pseudoaneurysm previously demonstrated.

At 7 weeks postoperatively, however, he was referred from an outside hospital for left thigh pain, swelling, and symptomatic anemia (tachycardia). The patient reported that he had received 2 blood transfusions at the outside hospital in the previous 3 weeks since his follow-up. Hemoglobin at time of presentation was 8.8 mg/dL. Due to continued need for transfusions, further investigation at the outside hospital was performed. A CT scan of his left thigh from the outside hospital was suspicious for a pseudoaneurysm (Figure 6). Diagnosis was confirmed at our institution by conventional angiography, which identified 13 mm pseudoaneurysm in the left profunda femoris artery. The patient underwent embolization of the pseudoaneurysm via one coil and Gelfoam (Pfizer, Wayne, PA) without issue (Figures 7, 8, and 9). Following the procedure the patient's pain improved and hemoglobin levels stabilized. He underwent a left thigh hematoma evacuation and drainage the following day to remove the large thigh hematoma, following which he experienced no further complications. He was discharged to his rehabilitation facility following the procedure.

## 3. Discussion

This case report is, to our knowledge, the only reported profunda femoris pseudoaneurysm following a revision total hip arthroplasty. It is only the 3rd reported case in any total hip arthroplasty [5, 6]. Despite being a rare complication, delayed or undetected vascular injuries following any procedure may be significant. Complications from vascular injuries include intraoperative or perioperative bleeding, critical limb ischemia, pain, pulsating mass or swelling, and hematoma development [4]. Additionally, the relationship between hematoma and early infection after THA has been well documented [7]. Hematoma acts as a source of infection and, if fascial defects are present, can quickly become a deep infection [7]. Postoperative THA infections have been associated with a higher number of hospitalizations, total number of hospital days, total number of operations, and increased hospital costs in both the inpatient and the outpatient settings [8]. Therefore, hematoma development associated with presentation of pseudoaneurysm needs to be identified promptly.

The lower extremity vasculature around the hip is complex, with many branches crossing the surgical field. The common femoral artery enters the leg through the femoral triangle, where it divides into the profunda femoris and superficial femoral arteries (SFA). The SFA gives off further 5 branches (superficial circumflex iliac, superficial epigastric, superficial external pudendal, deep external pudendal, and descending genicular artery). The profunda femoris artery runs between the pectineus and the posterior side of the adductor longus [9]. It gives off the lateral femoral circumflex artery, medial femoral circumflex artery (MFCA), and adductor perforating arteries as it traverses the thigh [10]. The MFCA gives off further 5 branches (superficial, ascending, acetabular, descending, and deep arteries) that provide the majority of the blood supply to the femoral head [11].

Profunda femoris pseudoaneurysms have been previously reported in femoral shaft fractures [4, 12, 13] and hip core decompression [14], with slightly more prevalence than THA. The more common arteries injured during total hip arthroplasty include external iliac, common femoral, and femoral circumflex arteries [4, 15, 16]. Causes of PFA lesions in other studies were suspected to be from an avulsion injury likely during manipulation [17], injury during placement of hardware for a DHS [4], and violation during drilling for placement of an intramedullary nail [13]. Different causes of pseudoaneurysm of all vessels following THA have been reported in literature, including retractor placement [12, 17], screw encroachment [4, 14], drill encroachment [12, 13], reduction techniques [17], removal of hardware [4], and cement exothermic reactions [17].

Cases reported presentation of pseudoaneurysm at the time of revision surgery for various reasons [17, 18], including pain [18], swelling [4], bleeding [5, 14, 16], vascular insufficiency [4], and chronically draining sinus development [4, 16]. Previous studies have reported unresolving pain following surgery in the presentation of pseudoaneurysms in other locations [4, 12, 13, 15]. This has been proposed as one of the possible causes of the patient's pain at initial presentation. Average detection time in these patients ranged from 4 months to 6 years after index procedure [4, 15, 16]. Further need for transfusions postoperatively was cited as the presenting or primary complaint in previous case reports [12, 14, 17]. The common delayed presentation can be attributed to the nature of how pseudoaneurysms form by incomplete vasculature damage and subsequent progressive expansion or embolism at the site of injury.

In our patient the cause of this pseudoaneurysm remains unknown. Placement of the femoral retractors, hip positioning during dislocation, and delayed presentation from the initial, anterior approach THA have been proposed.

During a posterior approach, retractors are often placed medially and laterally around the proximal femur after the hip is dislocated. Retractor placement medially around the femoral neck during a posterior approach may compress or injure the PFA as it travels between the pectineus and adductor longus after branching laterally from the common femoral artery [9]. Alternatively, previous case reports have shown damage to the common femoral artery with retractor placement on the anterior acetabulum [19].

The PFA injury may have occurred during the anterior approach at the time of his index THA. The patient's surgery was originally performed on the HANA table (Mizuho OSI, Union City, CA). A standard interval between the tensor fascia lata (TFL) and sartorius muscle was used. For surgeons who are perhaps unfamiliar with the approach, it is possible to dissect the plane too far medially and encroach onto the neurovascular bundle [20]. Similar to the posterior approach, retractor placement around the proximal femur may have caused vascular damage. During preparation of the femur on the HANA table, a hook is placed posteriorly behind the proximal femur and the leg is brought into hyperextension. Once hyperextended, the hook around the posterior aspect of the proximal femur is activated by a foot pedal, which displaces the proximal femur anteriorly, further accentuating the hyperextension of the hip and elevating the femur for better visualization and preparation of the femoral canal. In this position, the PFA, an anteriorly based structure, is placed on stretch. This position has been documented to cause lateral femoral cutaneous nerve palsies [21] and theoretically could cause similar stretch injuries to local vasculature. Other studies have shown that stretching of vascular structures, whether on a HANA table or during dislocation or manipulation, may result in an avulsion-type injury to vasculature structures in the area [17].

The early failure of the primary THA is difficult to explain, as there were no issues regarding alignment or infection at the time of revision surgery. Multiple papers have stated that heavier (BMI > 25) males are at greater risk of aseptic loosening of THA, particularly the femoral stem, in the early postoperative period [22, 23]. Alternatively, the PSA may have been present from the initial surgery (as mentioned above), and the regional bleeding in the area may have led to early aseptic loosening. Studies have shown that regional hematomas can lead to bone absorption and osteolysis around THA implants [24].

## 4. Conclusion

Vascular injury during THA is a rare but serious complication. Despite their infrequent occurrence, the clinician should always consider a vascular injury in the setting of post-op complications. High suspicion in the setting of unresolving postoperative pain or need for blood transfusions may allow for earlier diagnosis and avoidance of serious limb-threatening complications. Care during the procedure for accurate retractor placement and careful manipulation may help avoid serious complications.
